# Effects of microgravity on neuromuscular control of the spine: a protocol for a systematic review and meta-analysis

**DOI:** 10.1136/bmjopen-2024-098172

**Published:** 2025-06-20

**Authors:** Valter Devecchi, Michail Arvanitidis, Deborah Falla

**Affiliations:** 1 Centre of Precision Rehabilitation for Spinal Pain, School of Sport, Exercise and Rehabilitation Sciences, University of Birmingham, Birmingham, UK

**Keywords:** Spine, Rehabilitation medicine, NEUROPHYSIOLOGY

## Abstract

**Abstract:**

**Introduction:**

As spaceflight missions become more frequent and prolonged, the effects of microgravity on the musculoskeletal system represent a critical concern for astronauts’ health given their increased risk of spinal pain and injury. A better understanding of the adaptations induced by microgravity on neuromuscular control of the spine is essential to guide the development of effective countermeasures. Thus, this systematic review will aim to investigate the effects of microgravity on the neuromuscular control of the spine.

**Methods and analysis:**

This protocol has been developed following the Preferred Reporting Items for Systematic Review and Meta-Analysis Protocols. MEDLINE, EMBASE, CINAHL, Web of Science, PubMed, grey literature and specialised space research resources will be searched from inception up to December 31, 2024. Screening processes, data extraction and risk of bias assessment will be conducted by two independent reviewers. Studies investigating the acute and long-term effects of microgravity on neuromuscular control of the spine will be included. Studies investigating spaceflight conditions or other protocols simulating microgravity, such as parabolic flights, dry immersion and long-term bed rest, will be considered eligible. Non-randomised studies of intervention with before-and-after design will represent the main studies of interest, and their risk of bias will be evaluated with the Risk Of Bias In Non-randomised Studies-of Interventions tool. Random-effect meta-analyses will be conducted for quantitative synthesis when clinical and methodological consistency is ensured. The certainty of evidence will be evaluated using the Grading of Recommendations, Assessment, Development and Evaluation guidelines.

**Ethics and dissemination:**

As this systematic review is based on previously published studies, no ethical approval is required. The findings will be disseminated through publication in an international peer-reviewed journal and presented at conferences. All data relevant to the study will be included in the article or uploaded as supplementary information.

**PROSPERO registration number:**

CRD42024608544.

**STRENGTHS AND LIMITATIONS OF THIS STUDY**

- This will be the first systematic review to provide a comprehensive summary of the evidence on both the acute and long-term effects of microgravity on neuromuscular control of the spine.
- To ensure high-quality reporting, this protocol is presented following the Preferred Reporting Items for Systematic Review and Meta-Analysis for Protocols.
- Subgroup analyses will be conducted based on the specific microgravity protocol used, enabling comparisons between adaptations observed under simulated microgravity conditions and those resulting from actual spaceflights.
- Studies reported in languages other than English, Italian, Greek or Spanish will not be included.

## Introduction

 The exploration of space has entered a new era characterised by increased frequency, duration and investment in spaceflight missions.^1^ However, with this increase in mission number and length, it is essential to consider the physiological impacts of microgravity on the human body to ensure astronaut health and mission success. Several longitudinal studies assessing astronauts before and after spaceflight have demonstrated the negative impact of microgravity on human physiology, affecting the cardiovascular,^2^ immune,^3^ endocrine^4^ and notably, musculoskeletal systems.^5 6^ Due to microgravity and a reduction of mechanical loading, astronauts experience a disruption of the homeostatic balance of musculoskeletal structures which are typically present under Earth’s gravitational conditions.^7^ For example, significant changes have been reported in the spine after exposure to microgravity, including a reduction in bone density,^6^ hyperhydration of the intervertebral discs,^8^ muscle atrophy^5 9^ and a decrease in muscle strength,^9^ especially of those muscles with antigravitational roles. Collectively, these changes pose significant challenges to astronauts after spaceflight, given that the spine is essential for multiple antigravity functions, including maintaining postural balance, supporting limb movements and facilitating voluntary actions like bending and lifting. These alterations might explain the higher rate of spinal pain and injury among astronauts, with data showing an incidence of intervertebral disc herniation 21.4 and 2.8 times higher for the cervical and lumbar spine, respectively, compared with a control population.^8^

Despite the well-documented structural changes in the spinal region after spaceflight,^6 8 9^ evidence on the impact of acute and long-term exposure to microgravity on the neuromuscular control of the spine remains less clear. Specifically, no systematic reviews have been conducted to identify the changes in motor behaviour of the spine during voluntary or postural tasks, as well as during neurophysiological tests aimed at assessing corticospinal excitability and vestibular function. Understanding these changes is essential, as evidence from terrestrial conditions has shown that having altered neuromuscular control of the spine can lead to injury, pain recurrence and poor recovery after an acute episode of pain.^10^,^12^ For example, a longitudinal study showed that athletes with altered recruitment of their trunk muscles during spinal perturbations were at higher risk of low back injuries over a 3-year follow-up.^12^ Another study revealed that the low corticospinal excitability of back muscles after an acute episode of low back pain predicts high pain intensity and disability at 6 months.^11^ Interestingly, interventions targeting these neuromuscular adaptations resulted in positive outcomes, highlighting the importance of addressing neuromuscular control during rehabilitation programmes.^13 14^

Given the insights from terrestrial studies, it is crucial to understand how microgravity affects neuromuscular control of the spine in astronauts. Identifying neuromuscular adaptations during and after exposure to microgravity can inform strategies for developing countermeasures offered pre, during and post flight. Additionally, integrating knowledge of observed neurophysiological changes with structural changes can enhance our understanding of the overall response of the musculoskeletal system to microgravity. This is timely, as current interventions aiming to restore the mechanical function of the musculoskeletal system have provided benefits for passive structures like bone density, but these countermeasures have shown limited effectiveness in addressing muscle function.^9^ Thus, the aim of this systematic review is to investigate the effects of microgravity on the neuromuscular control of the spine. By summarising current knowledge, we hope to inform the development of effective countermeasures to mitigate the adverse effects of microgravity on spinal health and function.

## Methods

This protocol was developed in accordance with the Preferred Reporting Items for Systematic Review and Meta-Analysis Protocols (PRISMA, see online supplemental file 1)^15^ and the second edition of the Cochrane Handbook for Systematic Reviews of Interventions.^16^ The current protocol has been prospectively registered on PROSPERO (CRD42024608544). The findings from this systematic review and meta-analysis will be reported following the PRISMA 2020 statement.^17^

This systematic review will address the following question: ‘What are the acute and long-term effects of microgravity on neuromuscular control of the spine?’. Additionally, two secondary review questions will be considered:

2.a Do neuromuscular control changes remain after exposure to microgravity? If so, for how long?

2.b Do neuromuscular control changes differ based on the microgravity protocol used?

### Eligibility criteria

The inclusion and exclusion criteria for the studies in this review are guided by the PICOS framework (P: Population; I: Intervention; C: Comparator; O: Outcome(s); S: Study design).^15^

#### Population

The population of interest for this systematic review is healthy adults over 18 years of age.

#### Intervention

Studies investigating neuromuscular changes of the spinal region to microgravity will be included. We will consider studies involving exposure to microgravity or protocols simulating microgravity conditions as suggested by previous studies.^18 19^ The microgravity protocols to be included are spaceflight, parabolic flights with gravity less than or equal to 0.25 g, long-term bed rest, head-down tilt and dry immersion.^18 19^ For parabolic flight studies with multiple gravity-level conditions, only data from the lowest gravity level will be considered to better represent microgravity.

#### Comparators

Assessments conducted on the same participants during normal gravity conditions prior to microgravity exposure will serve as the comparator, using a within-subject design. This approach allows for direct comparison of neuromuscular control changes due to microgravity exposure while controlling for individual variability. To facilitate the description of the comparisons of interest, we will consider four specific time points in the evaluation of neuromuscular control.

- Baseline condition (BASE): assessment conducted before exposure to any microgravity protocol. If multiple baseline assessments are available, the one closest in time to the microgravity exposure will be used for further analysis. If only the average value of the baseline assessments is provided, this average will be used.
- Acute microgravity exposure (AµG): refers to assessments conducted during microgravity exposure and within the first 24 hours.
- Long-term microgravity exposure (LTµG): refers to the assessment conducted after microgravity exposure (within 48 hours) in a normogravity condition. When multiple assessments after microgravity are performed, the one closest in time to microgravity exposure will be considered.
- Recovery post-microgravity exposure (POSTµG): refers to assessments conducted at least 48 hours after returning to normogravity following microgravity exposure. When multiple assessments at different time points are present, these will all be extracted and used for further analyses.

Different comparisons of interest will be considered based on the time points reported and the review questions. If a study did not assess all time points (eg, only the acute or the long-term effect of microgravity), it will still be included, and only the evaluated time points will be considered. To address the primary review question, the BASE will be compared with the conditions of exposure to microgravity (ie, AµG and LTµG) to assess the immediate and sustained effects of microgravity on neuromuscular control of the spine. For the secondary review question *2*.a, neuromuscular control assessed in the period of recovery POSTµG will be compared with baseline (BASE) to determine if changes persist over time, and based on the included studies, different time points (eg, 1 week, 1 month or 1 year) will be considered. For the secondary review question 2.b, comparisons will be made between spaceflight and other microgravity protocols, such as spaceflight versus bed rest, to evaluate if changes in neuromuscular control of the spine differ based on the type of microgravity exposure.

#### Outcomes

The outcomes of interest for this review consist of the changes in neuromuscular control of the spine. Specific outcome measures include spinal kinematics, muscle activity, torque production and kinesthesia. These outcomes will be evaluated during voluntary tasks, static postures and responses to perturbations, including reactions to external or internal perturbations like mechanical indentation, moving platforms or rapid arm movements. Furthermore, we will also consider changes in the response to neurostimulation directly affecting the motor output of the spinal region, like the response of spinal muscles to vestibular or motor cortex stimulation. Studies focusing solely on mechanical characteristics of the spinal region, such as muscle tone, bone density and muscle composition will not be considered, as these aspects are not directly related to neuromuscular control and have been extensively investigated in previous work. Regarding the body region of interest, we will include assessments of movement, torque, or kinesthesia evaluated along the entire spine, as well as the activation of any muscle with a direct biomechanical action on the spine.

#### Study design

The study design of interest is a within-subject repeated measures design. Therefore, randomised trials (crossover design only) and non-randomised studies of interventions (NRSI with repeated measures) will be included. However, if a trial includes a control group receiving no intervention, the study will still be included, and for the subgroup not receiving the intervention, data on neuromuscular control assessed before and after microgravity exposure will be extracted.

#### Language

Studies will be included when reported in English, Italian, Greek or Spanish. Articles with relevant titles and abstracts reported in other languages will be excluded and listed in an appendix.

### Information sources

A single reviewer (VD) will conduct the search from inception up to 31 December 2024. The databases to be searched include MEDLINE (via OVID), EMBASE (via OVID), and CINAHL (via EBSCO). Additionally, specific internet sites will be searched, such as PubMed and Web of Science (Clarivate Analytics), along with specialised space research resources like the NASA Technical Reports Server, European Space Agency (ESA) Library, International Space Station Research and Committee on Space Research (COSPAR) websites. Hand-searching will be performed based on the scoping search results, focusing on journals relevant to this review topic—specifically, Acta Astronaut, Aerospace Medicine and Human Performance and NPJ Microgravity. Furthermore, the reference lists of included studies and relevant reviews will be checked. To reduce the risk of publication bias, grey literature databases like ProQuest and Ethos will be searched. Relevant authors in the field will also be contacted to obtain information about unpublished data or ongoing projects.

### Search strategy

Date, region, species (human vs non-human studies) and language will not be used as restrictions for the search, as the use of search filters is advised with caution because they have been demonstrated to potentially lead to publication bias.^20^ The search strategy and process have been developed and conducted by one reviewer (VD) with the assistance of an experienced librarian. The search will focus on three main concepts, including intervention, outcome of interest and body region connected as follows:

(microgravity) AND (“neuromuscular control”) AND (spine)

where “microgravity” identifies the interventions commonly used to reproduce or simulate microgravity exposure in studies (eg, “space flight”, “parabolic flight”, “weightlessness”, etc); “neuromuscular control” refers to the outcomes of interest (eg, kinematics, “muscle activity”, proprioception, etc); and “spine” refers to the body region of interest, including all portions of the spine such as “cervical”, “thoracic”, “lumbar”, etc. Terms within the same concepts will be separated by the Boolean operator “OR”. An example of a search strategy on MEDLINE (OVID interface) is provided in table 1. The search will be conducted using both subject headings (MeSH) and free-text terms to ensure a comprehensive retrieval of relevant studies. The search strategy will be adapted for different databases, but consistency will be ensured.

**Table 1:** Search strategy on MEDLINE (Ovid interface)

Intervention	exp Weightlessness/ or exp hypogravity/ or space flight/ or bed rest/ or head-down tilt/ or (microgravity or Weightlessness or hypogravity or “Hypo gravity” or “Zero gravity” or “Low gravity” or “Zero-gravity” or “Near-zero gravity” or “No gravity” or “Reduced gravity” or “free fall” or “micro-G” or “minimal gravity” or spaceflight or “Space flight” or “Space Travel” or “Space exploration” or “near-zero G” or Subgravity or “null gravity” or “parabolic flight” or “bed rest” or “dry immersion” or “head down tilt”).mp
Outcome	Range of motion, Articular/ or Biomechanical Phenomena/ or exp proprioception/ or Movement/ or Locomotion/ or exp psychomotor performance/ or motor skill/ or muscle strength/ or muscle strength dynamometer/ or electromyography/ or postural balance/ or muscle fatigue/ or physical endurance/ or muscle contraction/ or isometric contraction/ or isotonic contraction/ or exp evoked potentials, motor/ or transcranial magnetic stimulation/ or exp Physical Functional Performance/ or exp “Task Performance and Analysis”/ or (“motor control” or “motor coordination” or “corticospinal excitability” or “motor evoked potential” or “motor evoked potentials” or TMS or “transcranial magnetic stimulation” or “corticospinal drive” or “corticospinal function” or “vestibular stimulation” or “galvanic stimulation” or “Sensorimotor control” or “somatosensory function” or Proprioception or “joint position” or “joint reposition” or “position sense” or Kinesth* or “motor skill” or “muscle activation” or “muscle activity” or “muscle recruitment” or “muscle contraction” or Electromyo* OR emg OR “Neuromuscular control” OR “motor function” OR “motor output” OR Kinematic* OR Kinetic* OR “motor unit” OR “postural control” OR “postural balance” OR “movement analysis” OR “analysis of movement” OR “motion analysis” OR “movement control” OR “range of motion” OR “range of movement” OR “muscle force” OR “muscle strength”).mp
Body region	exp spine/ or exp torso/ or exp neck/ or exp abdominal muscles/ or exp back muscles/ or neck muscles/ or (spine or Back or Lumbar or Thoracic or Trunk or Cervical or Neck or Lumbopelvic or Lumbosacral or Spinal or Dorsal or Torso).mp
4.	1 AND 2 AND 3

### Data management

Data management, including the collection of citations, abstracts and full texts of relevant studies, will be performed using EndNote V.21 (Clarivate Analytics). During the search process, studies will be uploaded and duplicates removed by a single reviewer (VD). Once the search is complete, the list of studies will be imported into Covidence, a web-based platform used by two reviewers (VD and MA) to facilitate the screening process. Full texts of records that are potentially eligible will be uploaded and stored in Covidence, where their screening will also be conducted.

### Selection process

The screening process will be performed by two independent reviewers (VD and MA). In the initial stage, titles and abstracts will be evaluated using a piloted screening tool that aligns with the eligibility criteria and the primary review question. Any disagreement between reviewers will be resolved through discussion. If consensus cannot be reached, a third reviewer (DF) will be consulted to mediate the decision. Following this, full-text articles of potentially eligible studies will be collected and subjected to a second round of screening. Again, if the two reviewers disagree, the third reviewer (DF) will provide arbitration. Throughout both screening stages, the level of agreement between the two reviewers will be assessed using the kappa statistic, where kappa values between 0.60 and 0.74 are considered to indicate substantial agreement, and values of 0.75 or higher represent excellent agreement.^21^ Finally, the study selection process will be illustrated and summarised using the PRISMA flow diagram.^15^

### Data extraction process

Data extraction will be performed by one reviewer (VD), followed by a verification of accuracy conducted by a second reviewer (MA). A data extraction template will be developed by two reviewers (VD and MA) using Covidence software. Disagreements between reviewers will be resolved by discussion. Where necessary, a third reviewer (DF) will be consulted to mediate. When data related to the outcome of interest are not presented in a format suitable for data synthesis, they will be extracted from figures using WebPlotDigitizer. This software digitises data points from images and retrieves the necessary numerical information. WebPlotDigitizer has demonstrated high intercoder reliability and validity when the extracted data are compared with the original data.^22^ When the methods and results of primary studies are unclear or if unpublished information is needed for data synthesis, the authors will be contacted. Authors will be contacted no more than twice; an initial email will be sent, and if no response is received within fifteen days, a follow-up email will be sent. If there is no reply to the reminder email, the data will be considered irretrievable. When multiple records of the same study are identified, they will be collated, and the record with the most comprehensive description and data reporting will be selected as the primary source for data synthesis.^23^

### Data items and outcome prioritisation

The variables of interest are selected based on the PICOS framework, as well as the requirements for risk of bias (RoB) assessment and data synthesis. Therefore, the data extracted will include information relating to:

- Report identification features (title, first author and year).
- Study design and setting.
- Sample characteristics (age and gender).
- Interventions delivered (ie, microgravity protocol with brief description) and duration of exposure to microgravity to differentiate between acute and long-term effects.
- Comparators, represented by assessment conducted at baseline and during or after exposure to microgravity.
- Outcomes of interest and outcome measurement tools.
- Performed task.
- Available results, including p value, effect size, mean change, SD of the change.
- Funding information and conflict of interest.

After extraction, this information will be summarised in the ‘Characteristics of included studies’ table. Data necessary for synthesis will include the number of participants considered for the analyses, time points of assessment after microgravity exposure (to address review question 2.a), summary statistics (eg, means and SD) for each condition investigated (BASE, AµG, LTµG, POSTµG) and effect estimates between these conditions. In the ‘summary of findings’ table, results will be grouped by outcome domain and spinal region investigated. This will facilitate the comparison of results across different types of microgravity protocols, addressing review question 2.b.

### Risk of bias

Two independent reviewers (VD and MA) will evaluate the RoB. Any disagreements will be resolved through discussion, and if necessary, a third reviewer (DF) will be consulted for arbitration. This systematic review will consider findings from analyses with a repeated measures design; therefore, RoB will be assessed using the Risk Of Bias In Non-randomised Studies-of Interventions (ROBINS-I) tool for uncontrolled before-and-after designs.^24^ The assessment will cover biases related to confounding, participant selection, classification of interventions, deviation from intended interventions, missing data, measurement of outcomes and selection of the reported result.^24^

The assessment resulting from the ROBIN-I consists of a domain-based approach, which is preferred over checklists or scales that lead to a summary of multiple components into a single number. Specifically, the ROBIN-I categorises the RoB judgement as low, moderate, serious and critical.^24^ In addition to the source of information supporting the RoB judgement, a graphical summary of RoB for each domain will be created using R software.^25^ Following the previously described process, authors of the included studies will be contacted up to two times to clarify any ambiguous information. If no response is received, the RoB for that specific domain will be considered as ‘unclear’. Identified RoB will not exclude a study from data synthesis, and, if a meta-analysis is conducted, overall risk of bias will be presented for each individual study. Results from all included studies will be presented, and a narrative discussion of the RoB will be reported. Furthermore, the RoB assessment will inform the quality of evidence for each outcome domain.

### Data synthesis

Quantitative syntheses will be performed for the primary review question and the secondary review question 2.a. Due to the designs of the included studies, effect estimates will be calculated using within-subject change scores, which involve normalising the mean change between conditions by the SD of that change. When the effect estimates are not provided, these will be derived from the available information, following the guidelines outlined in the Cochrane Handbook and other methodological sources.^16 26^

Quantitative synthesis will be performed when there is methodological consistency across the selected studies. Specifically, meta-analysis from two or more studies will be performed to address the primary review question and secondary review question 2.a when there is homogeneity regarding the outcome, body region investigated, and time point considered (ie, AµG, LTµG or POSTµG). Effects estimated from the comparisons of interest will be extracted and reported in the ‘Main findings’ table. Statistical heterogeneity in quantitative synthesis will be evaluated using the I^2^ statistic and the χ^2^ test. When studies demonstrate methodological consistency but use different microgravity protocols, they will be included in quantitative synthesis and a subgroup analysis will be used to address the secondary review question 2.b. We hypothesise that such prespecified subgroup analysis will partially address the substantial statistical heterogeneity (ie, I^2^>50%) that might exist across studies when pooled together. A quantitative synthesis will be conducted using a random-effects model with the inverse-variance method, as recommended by the Cochrane Back and Neck Group.^27^ This approach is selected due to the heterogeneity of outcome measures, microgravity protocols, and the inclusion of NRSI. While the random-effects model statistically accounts for heterogeneity, it does not eliminate it.^28^ Therefore, as recommended^28 29^ and done previously,^30^ additional measures of heterogeneity will also be explored, including τ², which provides a quantitative estimate of between-study variance, calculated using the restricted maximum-likelihood estimator. When a sufficient number of studies are included (approximately 10), prediction intervals will also be calculated. These intervals offer valuable insights into the expected range of true effects in future comparable studies, providing a more interpretable summary of the spread of underlying effects within the included studies.^28 29^ This comprehensive approach will enhance the robustness and clinical relevance of the findings.

Effect estimates for continuous outcomes will be reported as standardised mean differences of the changes, with their variances expressed using 95% CIs. Although dichotomous outcomes are rarely used to describe the primary outcomes in this review, any such findings will be summarised using risk ratios and 95% CI. When studies provide effect estimates adjusted for significant confounding variables that might influence the results, these adjusted estimates will be extracted from the study analyses, and the confounders considered will be reported. Forest plots and results from quantitative syntheses will be graphically presented and summarised using R software. Overall findings will also be presented using systematic narrative synthesis and structured tabulation, reporting the neuromuscular control changes for specific outcome, body region and microgravity protocol. When meta-analysis is precluded, forest plots will still be used to provide a graphical description of the results. This will facilitate the interpretation of the direction of effect supporting the vote-counting synthesis, as well as identify potential sources of heterogeneity. The presentation of findings will not be restricted by the risk of bias.

### Sensitivity analyses

Given the anticipated high heterogeneity, sensitivity analyses will be conducted to ensure the robustness of our findings and to assess the impact of individual studies on the overall results.^28^ This will include excluding each study in turn to evaluate the consistency of the meta-analysis results and determine the influence of individual studies on heterogeneity and pooled effect sizes. Additionally, recognising that outliers and influential cases may compromise the validity and robustness of meta-analysis conclusions, we will quantitatively identify potential outliers using deletion diagnostics adapted from linear regression. This analysis will be conducted using the ‘influence’ function from the ‘metafor’ package in R.^31 32^ Studies identified as being of poor methodological quality will be removed to examine their impact on the results. This comprehensive approach to sensitivity analyses will ensure the robustness of our meta-analysis and provide a deeper understanding of the influence of individual studies on the overall findings.

### Meta-biases

The risk of publication bias will be evaluated by searching grey literature databases and dissertations. Funnel plots will be generated and inspected for potential asymmetries which are signs of publication bias, along with the Egger’s regression test.^33^ The discrepancy between planned analysis and reported findings will be assessed when multiple sources of information from the same study are available. Also, study authors will be contacted when results that should be reported are completely unavailable.

### Confidence in cumulative estimate

The Grading of Recommendations Assessment, Development and Evaluation (GRADE) approach and its associated guidelines will be used to assess the certainty of the body of evidence.^34^ The certainty of evidence will be assessed regarding the acute and long-term effects of microgravity on neuromuscular control of the spine. Hence, evidence from the comparisons between BASE and AµG or LTµG will be considered and presented separately. Moreover, the certainty of evidence will be evaluated for each outcome domain of interest, and results will be presented in the ‘Summary of findings’ table which will present information on population (number of participants and number of studies), microgravity protocol used, spinal region investigated, GRADE assessment and overall certainty of evidence.^35^ Judgements on the quality of evidence will be supported by explanatory reasons. Overall, the GRADE system identifies four levels of evidence, including ‘high’, ‘moderate’, ‘low’ and ‘very low’.^36^ The level of certainty can be downgraded based on the assessment of five domains, including study limitations, inconsistency, indirectness, imprecision and publication Bias.^35^ When a large effect estimate is present, the level of evidence is upgraded.^35^ Also, a dose-response gradient showing a larger effect based on the level of microgravity recreated (eg, 0 g vs 0.25 g) or the length of exposure will upgrade the level of evidence. The presence of inconsistency across studies will be evaluated through prespecified subgroup analysis, considering the type of microgravity protocol and risk of bias.

### Patient and public involvement

Patients or the public were not involved in the design, conduct, reporting or dissemination plans of our research.

## Discussion

The findings from this systematic review will reveal if and what adaptations in the neuromuscular control of the spine are caused by microgravity. When integrated with the structural changes of the spine already summarised in previous work,^6 8 9^ these results can provide new insights into why astronauts are at greater risk of spinal pain and injury after exposure to microgravity. For example, identifying adaptations during acute and long-term exposure to microgravity may uncover changes in neuromuscular control that need to be addressed with specific interventions during and after space missions. Furthermore, assessing the effects several days and months post-microgravity can identify adaptations that persist despite returning to normal gravity. These persistent changes will require particular attention, as baseline neuromuscular control might be restored only through specific interventions.

By considering the neurophysiological characteristics of different microgravity protocols, it will be possible to understand if specific types of microgravity exposure result in different neuromuscular adaptations of the spine. Demonstrating which specific microgravity protocol best replicates the features of neuromuscular adaptation observed after spaceflight will help researchers choose the most appropriate protocol for future studies.

Findings from this systematic review might be affected by some limitations. Specifically, including studies with different research designs, microgravity protocols and outcome measures is likely to generate substantial heterogeneity and preclude quantitative synthesis. Furthermore, microgravity research typically relies on small sample sizes, increasing both imprecision and the risk of publication bias. Finally, we will restrict eligibility to articles published in English, Italian, Greek or Spanish, which may introduce language bias.

### Ethics and dissemination of results

This systematic review will be based only on previously published studies in which participants provided informed and voluntary consent. Therefore, ethical approval is not required. The findings will be presented at national and/or international conferences and submitted for publication in a peer-reviewed journal. All data relevant to the study will be included in the article or uploaded as supplementary information.

## Supplementary material

10.1136/bmjopen-2024-098172 online supplemental file 1
