# Supplementary material for: Effects of microgravity on neuromuscular control of the spine: a protocol for a systematic review and meta-analysis
Source: BMJ Open. 2025 Jun 20;15(6):e098172. doi: 10.1136/bmjopen-2024-098172 (PMC12182208; doi:10.1136/bmjopen-2024-098172)
Supplement: Supplementary file 2 [file bmjopen-2024-098172.reviewer_comments.pdf]

PEER REVIEW HISTORY

BMJ Open publishes all reviews undertaken for accepted manuscripts. Reviewers are asked to complete a checklist review form and are provided with free text boxes to elaborate on their assessment. These free text comments are reproduced below.

ARTICLE DETAILS

Title (Provisional)

The effects of microgravity on neuromuscular control of the spine: a protocol for a systematic review and meta-analysis

Authors

Devecchi, Valter; Arvanitidis, Michail; Falla, Deborah

VERSION 1 - REVIEW

**Reviewer**	**1**
**Name**	**Zhu, Qiaoqiao**
**Affiliation**	**Nanjing University**
**Date**	**13-Mar-2025**
**COI**	**None**

Overall, it is a well-written review protocol for examining the effects of microgravity on neuromuscular control of the spine; however, the limitations of this protocol were not included.

**Reviewer**	**2**
**Name**	**Kuo, Fang-Chuan**
**Affiliation**	**HungKuang University**
**Date**	**05-May-2025**
**COI**	**None**

●Line 1: Change " **Abstract** " to " **ABSTRACT** ".

●Line 16: Change " protocol " to " protocols ".

●Line 40: Change " **Introduction** " to " **INTRODUCTION** ".

●Line 51: Change " of " to " in ".

●Line 67: Change "low " to " the low ".

●Line 83: Change "**Methods** " to " **METHODS** ".

●Line 89: Change "question “*What* " to "question: “*What* ".

●Line 104-105: Change "gravity conditions " to "gravity-level conditions".

●Line 114-115: Change "an average value " to "the average value".

●Line 124: Change "be all " to "all be".

●Line 134: Change "different time points" to "at different time points".

●Line 144: Change "response " to "the response".

●Line 153: Change "randomized and non-randomized " to "randomised and non-randomised".

●Line 168: Change "reference" to "the reference".

●Line 180: Change "identify" to "identifies".

●Line 185: Change "search strategy" to "a search strategy".

●Line 188: Change "consistencies" to "consistency".

●Line 216: Change "Disagreement" to "Disagreements".

●Line 250: Change "Risk of Bias" to "Risk of bias".

●Line 270: Change "secondary review question" to "the secondary review question".

●Line 278: Change "when" to "When".

●Line 289: Change "non-randomized" to "non-randomised".

●Line 310: Change "risk" to " the risk".

●Line 326-327: Change "The discrepancy between planned analysis and reported findings will be assessed when multiple information from the same study is available." to " The discrepancy between planned analysis and reported findings will be assessed when multiple sources of information from the same study are available.".

●Line 334: Change "certainty" to " the certainty".

●Line 348-349: Change "Patients or the public were not involved in the design, or conduct, or reporting, or dissemination plans of our research" to " Patients or the public were not involved in the design, conduct, reporting, or dissemination plans of our research.".

●Line 350: Change "**Discussion**" to " **DISCUSSION**".

●Line 371: Change "**References**" to " **REFERENCES**

●**REFERENCES:** Corrected the manuscript according to the citation format of this journal. For example, Physical activity (PA) is important at all stages of life, including childhood,

adolescence and old age.[1 2]

1 An H-Y, Chen W, Wang C-W, et al. The Relationships between Physical Activity and Life Satisfaction and Happiness among Young, Middle-Aged, and Older Adults. Int J Environ Res Public Health 2020;17:4817.

2 Chaput J-P, Willumsen J, Bull F, et al. 2020 WHO guidelines on physical activity and sedentary behaviour for children and adolescents aged 5-17 years: summary of the evidence. Int J Behav Nutr Phys Act 2020;17:141.

VERSION 1 - AUTHOR RESPONSE

Reviewer: 1
Dr. Qiaoqiao Zhu, Nanjing University
Comments to the Author:
Overall, it is a well-written review protocol for examining the effects of microgravity on neuromuscular control of the spine; however, the limitations of this protocol were not included.

We thank the reviewer for the positive feedback. We expanded the Discussion section and we included a paragraph with the potential limitations of our study and methodology.

"Findings from this systematic review might be affected by some limitations. Specifically, including studies with different research designs, microgravity protocols, and outcome measures is likely to generate substantial heterogeneity and preclude quantitative synthesis. Furthermore, microgravity research typically relies on small sample sizes, increasing both imprecision and the risk of publication bias. Finally, we will restrict eligibility to articles published in English, Italian, Greek or Spanish, which may introduce language bias."

Reviewer: 2
Dr. Fang-Chuan Kuo, HungKuang University
Comments to the Author:
●Line 1: Change " Abstract " to " ABSTRACT ".
●Line 16: Change " protocol " to " protocols ".
●Line 40: Change " Introduction " to " INTRODUCTION ".
●Line 51: Change " of " to " in ".
●Line 67: Change "low " to " the low ".
●Line 83: Change "Methods " to " METHODS ".
●Line 89: Change "question "What " to "question: "What ".
●Line 104-105: Change "gravity conditions " to "gravity-level conditions".
●Line 114-115: Change "an average value " to "the average value".
●Line 124: Change "be all " to "all be".
●Line 134: Change "different time points" to "at different time points".
●Line 144: Change "response " to "the response".
●Line 153: Change "randomized and non-randomized " to "randomised and non-randomised".
●Line 168: Change "reference" to "the reference".
●Line 180: Change "identify" to "identifies".
●Line 185: Change "search strategy" to "a search strategy".
●Line 188: Change "consistencies" to "consistency".

●Line 216: Change "Disagreement" to "Disagreements".
●Line 250: Change "Risk of Bias" to "Risk of bias".
●Line 270: Change "secondary review question" to "the secondary review question".
●Line 278: Change "when" to "When".
●Line 289: Change "non-randomized" to "non-randomised".
●Line 310: Change "risk" to " the risk".
●Line 326-327: Change "The discrepancy between planned analysis and reported findings will be assessed when multiple information from the same study is available." to " The discrepancy between planned analysis and reported findings will be assessed when multiple sources of information from the same study are available.".

●Line 334: Change "certainty" to " the certainty".

●Line 348-349: Change "Patients or the public were not involved in the design, or conduct, or reporting, or dissemination plans of our research" to " Patients or the public were not involved in the design, conduct, reporting, or dissemination plans of our research.".

●Line 350: Change "Discussion" to " DISCUSSION".

●Line 371: Change "References" to " REFERENCES

●REFERENCES: Corrected the manuscript according to the citation format of this journal. For example, Physical activity (PA) is important at all stages of life, including childhood, adolescence and old age.1 2
1 An H-Y, Chen W, Wang C-W, et al. The Relationships between Physical Activity and Life Satisfaction and Happiness among Young, Middle-Aged, and Older Adults. Int J Environ Res Public Health 2020;17:4817.
2 Chaput J-P, Willumsen J, Bull F, et al. 2020 WHO guidelines on physical activity and sedentary behaviour for children and adolescents aged 5-17 years: summary of the evidence. Int J Behav Nutr Phys Act 2020;17:141.

We thank the reviewer for the feedback provided. We modified the manuscript accordingly.

VERSION 2 - REVIEW

**Reviewer**	**1**
**Name**	**Zhu, Qiaoqiao**
**Affiliation**	**Nanjing University**
**Date**	**05-Jun-2025**
**COI**	

Thanks for the revision, I have no more comments

**Reviewer**	**2**
**Name**	**Kuo, Fang-Chuan**
**Affiliation**	**HungKuang University**
**Date**	**23-May-2025**
**COI**	

●Line 370: Change " is " to " are ".
